# Effect of myrtol on chronic bronchitis or chronic obstructive pulmonary disease: A protocol for systematic review and meta-analysis

**DOI:** 10.1097/MD.0000000000020692

**Published:** 2020-07-10

**Authors:** Liyun Liu, Shuiqin Li, Yongcan Wu, Xiaomin Wang, Demei Huang, Caixia Pei, Fei Wang, Zhenxing Wang

**Affiliations:** a School of basic medical sciences, Chengdu University of Traditional Chinese Medicine, No. 1166 Liutai avenue, Wenjiang district; b Department of Respiratory Medicine, Hospital of Chengdu University of Traditional Chinese Medicine, No. 39 Shi-er-qiao Road; c Department of Geriatrics, Hospital of Chengdu University of Traditional Chinese Medicine, No. 39 Shi-er-qiao Road; d Department of Gastroenterology, Hospital of Chengdu University of Traditional Chinese Medicine, No. 39 Shi-er-qiao Road, Chengdu, Sichuan Province, People's Republic of China.

**Keywords:** chronic bronchitis, chronic obstructive pulmonary disease, meta analysis, myrtol, protocol, systematic review

## Abstract

**Background:**

The key to the management of chronic obstructive (CB) and chronic obstructive pulmonary disease (COPD) is to control symptoms of the disease and to prevent deterioration in the health of affected patients. Myrtol has been proved to be effective in treating the symptoms of patients with CB and COPD and preventing the deterioration in their health. However, there has been no systematic review of the efficacy and safety of myrtol in the treatment of CB or COPD. The purpose of this study is going to evaluate the effects of myrtol on the management of CB or COPD based on randomized controlled trials.

**Methods:**

Electronic literature and other ongoing studies will be searched before November 31, 2019. Randomized controlled trials that report the use of myrtol in the treatment of CB or COPD (in the absence and presence of concurrent treatments) will be selected for inclusion regardless of language. Primary outcomes will include cumulative numbers of exacerbation events and the number of days of disability including days in bed, days off work due to breathing complications, and days on which the participant was unable to undertake normal activities due to breathing complications. Study selection, data extraction, and deviation the derivation risk assessment will be carried out by 2 independent investigators. Meta-analysis will be carried out by the RevMan5.3 software.

**Results:**

The study will provide summary results for estimating the efficacy and safety of myrtol for future treatments of CB or COPD.

**Conclusions:**

This systematic review will determine if myrtol is an effective and a safe intervention on the symptoms and the prevention of exacerbation of CB or COPD.

**Ethics and dissemination:**

Ethical approval will not be required for this study because no identifying patient data will be used. The review will be published as an article or a conference presentation in a peer-reviewed journal.

**Registration:**

OSF registration number: DOI 10.17605/OSF.IO/PXRBV

## 1 Introduction

Chronic obstructive pulmonary disease (COPD) is a clinically preventable and treatable disease characterized by persistent respiratory symptoms and airflow constraints. It is currently the fourth leading cause of death in the world,^[1]^ but is projected to become the third leading cause of death by 2020.^[2]^ There are world-wide concerns about the high morbidity of COPD and its serious economic and social burdens.^[3,4]^ COPD results from an abnormal inflammatory response to exposure to harmful gases, as well as to particulate matter.^[2]^ Fatalities from COPD are high: an estimated 2 million people die from the disease each year in Asia, accounting for 2/3 of global COPD deaths.^[5]^ In China, more than 100 million people suffer from COPD.^[6]^

Although the National Institute for Health and Clinical Excellence (NICE) and the Global Initiative for Chronic Obstructive Lung Disease (GOLD) have recommended developing evidence-based treatments for COPD, including treatments using short- and long-acting bronchodilators, inhaled corticosteroids, and low-dose and sustained-release theophylline.^[2,7]^ Then these treatments have inevitable side effects,^[8,9]^ which have prompted some COPD patients to seek more alternative treatments. Therefore, it is extremely important to find alternative COPD treatments that lack undesirable side effects.

Myrtol is extracted from the leaves of plants of Myrtaceae and has been shown to have secretolytic, mucolytic, secretomotoric, and anti-inflammatory properties.^[10–13]^ In recent years, an increasing number of evidences revealed that mrytol has positive effect in chronic obstructive (CB) and COPD management, and has been widely used to treat CB and COPD.^[14–18]^ However, there is little evidence concerning its efficacy and safety for CB or COPD patients. Therefore, the aim of this study is to conduct a systematic review to explore the efficacy and safety of mrytol for CB or COPD.

### 1.1 Review objectives

Our objective is to provide a comprehensive systematic review and meta-analysis of the safety and efficacy of myrtol for patients with CB or COPD. The results of the research will provide evidence for clinicians in guiding the treatment of CB and COPD.

## 2 Methods

### 2.1 Protocol register

This study was registered on OSF. Registration number: DOI 10.17605/OSF.IO/PXRBV. Our meta-analysis will be based on the Preferred Reporting Items for System Review and Meta-analysis Protocols (PRISMA-P).^[19,20]^

### 2.2 Ethics

The study will be submitted to a peer-reviewed journal for eventual distribution in electronic and printed form. This study is a retrospective study of published literature and does not require ethical approval.

### 2.3 Data sources

#### 2.3.1 Electronic searches

Electronic literature searches will be conducted separately by 2 investigators (WYC and LLY) according to the syntax of each database and the principles of Population, Intervention, Comparison, Outcomes, and Study design (PICOS) in evidence-based medicine. We will search the following electronic database for studies on the treatment of CB or COPD uploaded since the establishment until November 31, 2019: PubMed/MEDLINE, EMBASE, the Cochrane Library, the Web of Science, Ovid, Scopus, the CNKI Database, the CBM database, the Wan Fang Database, and the VIP database.

Search terms to be considered for searches will include: “Gelomyrtol,” “Myrtol,” “myrtol standard,” “ELOM-080,” “COPD,” “chronic bronchitis,” “chronic obstructive pulmonary disease,” “Pulmonary Disease,” “bronchitis,” and “clinical trial.” The example of specific search for PubMed is shown in Table 1.

**Table 1:**
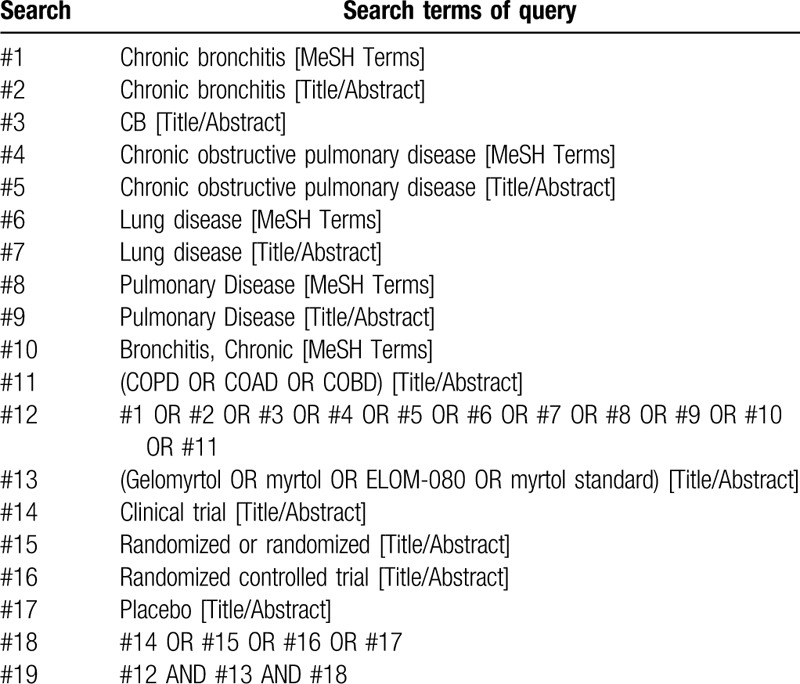
PubMed search strategies.

#### 2.3.2 Searching other resources

We will also retrieve unpublished data from ongoing studies in the NIH clinical registry Clinical Trials.Gov (https://www.clinicaltrials.gov/), the International Clinical Trials Registry Platform (ICTRP) (https://www.who.int/ictrp/en/), the Australian New Zealand Clinical Trials Registry (https://www.anzctr.org.au/), and the Chinese Clinical Registry (http://www.chictr.org.cn/index.aspx). Other publications, such as relevant systematic reviews and meta-analyses, will be reviewed to identify additional trials.

#### 2.3.3 Types of studies

This study will include prospective randomized controlled trials to evaluate the efficacy of myrtol in the treatment of CB or CPOD. We will exclude non-randomized controlled studies, animal experiments, cell experiments or literature review studies. There are no language restrictions in this study.

#### 2.3.4 Types of participants

Participants who meet the diagnostic criteria of CB or COPD will be included, including subjects with CB as defined by the British Medical Research Council, COPD as defined by the criteria of the American Thoracic Society, the Global Initiative for Chronic Obstructive Lung Disease (GOLD), the European Respiratory Society, or the World Health Organization(WHO).^[2,7,21]^ Patients with asthma, bronchiectasia, paeumonophthisis, and cystic fibrosis will be excluded.

#### 2.3.5 Types of interventions and controls

Randomized controlled trials with myrtol as the only treatment will be included. If the same intervention is performed in both the treatment group and the control group, a study will also be considered to evaluate the comprehensive effect of myrtol plus other interventions. For the control group, we will consider placebo or sham treatment, nonintervention and other interventions.

### 2.4 Outcome measures

#### 2.4.1 Primary outcomes

1. Cumulative numbers of exacerbations. Exacerbation was defined as an increase in cough and by volume and/or purulence of sputum. The primary outcome is the effect of myrtol on cumulative numbers of exacerbations during the treatment period.
2. Number of days of disability such as days in bed, days off work, and days on which the participant was unable to undertake normal activities.

#### 2.4.2 Additional outcomes

1. Measures of lung function, including forced expiratory volume in one second (FEV1), forced vital capacity (FVC), and peak expiratory flow rate (PEFR).
2. Adverse effects of treatment: Total number of adverse events.
3. Hospitalization and mortality.
4. Quality of life as measured by a tool validated in patients with COPD.

### 2.5 Data extraction

**Figure 1:**
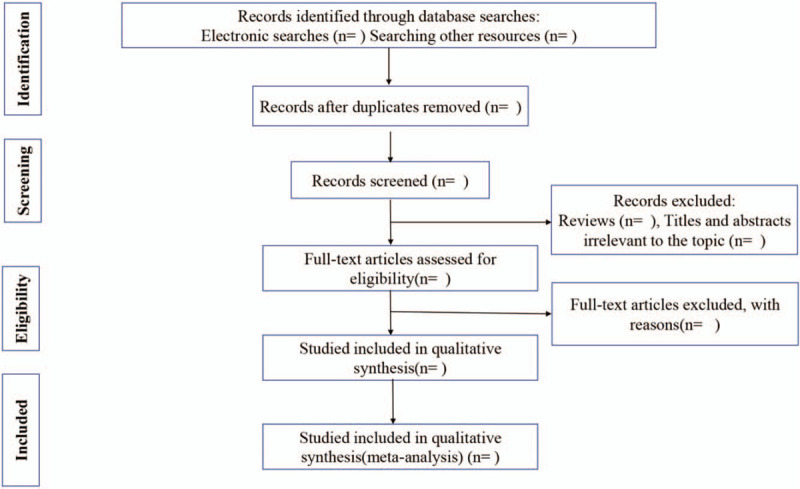
Flow diagram of the study selection process.

We will import the search results into Endnote X (V.9.0). Two investigators (PXC and HDM) will evaluate the retrieved studies based on inclusion criteria. They will conduct a preliminary screening of the title and abstract to exclude articles that do not meet requirements. Unmatched studies will be moved to the trash box in Endnote X (V.9.0). The reasons for the exclusion of selected studies will be recorded in an Excel table. The next step will be to read the full text and to evaluate the reason for inclusion based on the full text. Two investigators will examine the reference list to confirm possible missing studies. The results of the selection process will be cross-checked by 2 investigators. Any differences will be resolved through discussion and a consensus. A third investigator (LSQ) will arbitrate unresolved arguments. Each eligible study will be assigned a research ID using the following format: first author's last name + space + year of publication (for example, Wang 2019). The selection of studies will be summarized in a PRISMA flow diagram (Fig. 1).

Two investigators (WYC and LLY) will review the eligibility of each included study and extract data by using a standardized template. This standardized template will include the following information: article information (title, list of authors, year of publication, sample size, design, country, and sponsor), participant information (age, sex, number of samples in the group, and chronic bronchitis or COPD diagnostic criteria), intervention details (intervention therapy, comparator therapy, dosage, duration, administration route, and cointervention), and outcome details (measuring methods and adverse events). Any differences identified during data cross-checking will be resolved through discussion and the recommendation of a third investigator (LSQ).

### 2.6 Assessment of risk of bias in individual studies

The bias risk of each included study will be assessed by using Cochrane Collaboration's tools in randomized trials.^[22]^ Two investigators (LLY and WYC) will input the relevant information of each study into RevMan software.^[23]^ The quality of the methodology of each included study will be evaluated from 7 perspectives, including random sequence generation, allocation concealment, blinding of participants and personnel, blinding of outcome assessment, incomplete outcome data, selective reporting, and other potential sources of bias. The investigators will assess the risk of the 7 biased projects one by one, and grade the risk of each item into 1 of 3 levels as follows: high, low or unclear. Differences will be settled through discussion. A third investigator (LSQ) will be consulted, if necessary, to reach a consensus. If the essential information is lost or unreported, the authors of the original study will be contacted for the risk of deviation assessment. The assessment results will be displayed in the bias risk summary chart.^[22]^

### 2.7 Data synthesis

The differences of the intervention and control groups will be evaluated. The data of clinical studies will be input into Revman software (V.5.3) for data synthesis. The data will be synthesized and analyzed according to the level of statistical heterogeneity. If the study is homogeneous enough in terms of design and comparison, we will use a random effect model for meta-analysis. The heterogeneity will be tested by Cochrane *Q* test and *I*^2^ statistics. If *I*^2^ ≤ 50%, then the heterogeneity between multiple studies will be acceptable. When *I*^2^ > 50%, meta-analysis will not be carried out. We will try to find out the root causes of heterogeneity from various aspects and provide a narrative and qualitative summary.

Risk ratios with 95% CIs will be used to evaluate dichotomous outcomes. Continuous outcomes will be calculated by using the mean difference or standardized mean difference with 95% CIs. The mean difference will be applied if the same scale is used to estimate an outcome in different studies. The standardized mean difference will be applied for different measurement tools. Before statistical analysis, the units of the outcomes from the different studies will be transformed into the International System of Units.

Data may be missing from studies whether intentionally or unintentionally. We will contact the corresponding author of such studies for this reason. After the investigators confirm that the data was missing, the available data will be analyzed. The missing data will not be used in the preliminary analysis. However, the impact of the missing data will be assessed in the sensitivity analysis. The potential impact of the missing data on the final result of the review will be analyzed in the discussion.

#### 2.7.1 Subgroup analysis

If there is significant heterogeneity in the included trials, then we will conduct subgroup analysis according to the region of the studies, age, stage of the subjects, types of treatments, and different outcomes.

#### 2.7.2 Sensitivity analysis

Sensitivity analysis will be used to identify the robustness of the review conclusions, such as methodological weaknesses, missing data, and heterogeneity qualities. According to the situation of each clinical trial included in the integration, which factors can be considered to affect the integration results, so that it can be carried out according to the actual situation by excluding trials with high risk of bias or canceling each study on a case-by-case basis. And after performing the meta-analysis again, compare it with the previous meta-analysis.

#### 2.7.3 Assessment of publication bias

Funnel plots will be used to assess publication bias. If there is significant publication bias, then asymmetric funnel plots will be generated. More obvious asymmetries will indicate higher degrees of bias.

### 2.8 Grading of Recommendations Assessment, Development and Evaluation (GRADE) quality assessment

Online Grading of Recommendations Assessment, Development and Evaluation (GRADE) will be used to independently assess the quality of evidence for each outcome by the reviewers.^[24,25]^ The evidence level will be classified into four possible ratings as follows: very low, low, moderate, or high.

## 3 Discussion

At present, there are many treatment methods and drugs for COPD, including glucocorticoids, bronchodilators, antibiotics and so on, however these methods still cannot meet the needs of CB or COPD patients. The studies on myrtol in the treatment of CB or COPD increases at present, so it is necessary to evaluate the effect of myrtol on CB or COPD.

Myrtol has been proved to have the effects of anti-inflammation, dilution of mucus and so on. However, the efficacy and safety of myrtol in the treatment of chronic bronchitis or chronic obstructive pulmonary disease is not clear. Therefore, we believe that the results of this meta-analysis will provide some reference for clinicians and researchers.

### 3.1 Amendments

If there are any amendments in this protocol, we will give the date of each amendment, describe the situation of the change, and give the reason.

## Acknowledgments

We thank TopEdit (www.topeditsci.com) for its linguistic assistance during the preparation of this manuscript.

## Author contributions

**Conceptualization:** Liyun Liu, Yongcan Wu, Zhenxing Wang, Shuiqin Li, Fei Wang.

**Data curation:** Liyun Liu, Xiaomin Wang, Caixia Pei.

**Formal analysis:** Liyun Liu, Yongcan Wu, Caixia Pei, Xiaomin Wang.

**Funding acquisition:** Zhenxing Wang.

**Investigation:** Liyun Liu, Yongcan Wu, Xiaomin Wang, Fang Yang, Demei Huang.

**Methodology:** Liyun Liu, Yongcan Wu, Xiaomin Wang.

**Project administration:** Liyun Liu, Yongcan Wu, Zhenxing Wang, Shuiqin Li, Fei Wang.

**Resources:** Zhenxing Wang, Shuiqin Li.

**Software:** Caixia Pei, Demei Huang.

**Supervision:** Xuhong Yan, Liang Dong, Yinghong Liu, Degui Chang, Xujun Yu.

**Validation:** Liyun Liu, Yongcan Wu, Xiaomin Wang.

**Writing – original draft:** Liyun Liu, Shuiqin Li.

**Writing – review & editing:** Zhenxing Wang, Shuiqin Li, Fei Wang.
